# Comparative genome characterization of *Leptospira interrogans* from mild and severe leptospirosis patients

**DOI:** 10.5808/gi.21037

**Published:** 2021-09-30

**Authors:** Songtham Anuntakarun, Vorthon Sawaswong, Rungrat Jitvaropas, Kesmanee Praianantathavorn, Witthaya Poomipak, Yupin Suputtamongkol, Chintana Chirathaworn, Sunchai Payungporn

**Affiliations:** 1 Program in Bioinformatics and Computational Biology, Graduate School, Chulalongkorn University, Bangkok 10330, Thailand; 2 Division of Biochemistry, Department of Preclinical Science, Faculty of Medicine, Thammasat University, Pathum Thani 12120, Thailand; 3 Department of Biochemistry, Faculty of Medicine, Chulalongkorn University, Bangkok 10330, Thailand; 4 Research Affairs, Faculty of Medicine, Chulalongkorn University, Bangkok 10330, Thailand; 5 Department of Medicine, Faculty of Medicine Siriraj Hospital, Mahidol University, Bangkok 10700, Thailand; 6 Department of Microbiology, Faculty of Medicine, Chulalongkorn University, Bangkok 10330, Thailand; 7 Research Unit of Systems Microbiology, Chulalongkorn University, Bangkok 10330, Thailand

**Keywords:** genome annotation, leptospirosis, *Leptospira interrogans*, virulence factor genes

## Abstract

Leptospirosis is a zoonotic disease caused by spirochetes from the genus *Leptospira*. In Thailand, *Leptospira interrogans* is a major cause of leptospirosis. Leptospirosis patients present with a wide range of clinical manifestations from asymptomatic, mild infections to severe illness involving organ failure. For better understanding the difference between *Leptospira* isolates causing mild and severe leptospirosis, illumina sequencing was used to sequence genomic DNA in both serotypes. DNA of *Leptospira* isolated from two patients, one with mild and another with severe symptoms, were included in this study. The paired-end reads were removed adapters and trimmed with Q30 score using Trimmomatic. Trimmed reads were constructed to contigs and scaffolds using SPAdes. Cross-contamination of scaffolds was evaluated by ContEst16s. Prokka tool for bacterial annotation was used to annotate sequences from both *Leptospira* isolates. Predicted amino acid sequences from Prokka were searched in EggNOG and David gene ontology database to characterize gene ontology. In addition, *Leptospira* from mild and severe patients, that passed the criteria e-value < 10e^-5^ from blastP against virulence factor database, were used to analyze with Venn diagram. From this study, we found 13 and 12 genes that were unique in the isolates from mild and severe patients, respectively. The 12 genes in the severe isolate might be virulence factor genes that affect disease severity. However, these genes should be validated in further study.

## Introduction

Leptospirosis is a worldwide zoonotic disease that influences humans and animals worldwide [1]. It is a zoonosis caused by bacteria in the genus *Leptospira*. *Leptospira* can be clustered in three groups including pathogenic, intermediate pathogenic and saprophytic groups. The various clinical manifestations are caused by the pathogenic and intermediate groups, while the saprophytic group does not cause the disease in humans or animals [2]. Human leptospirosis can be acquired by contact with the urine of infected animals or soil and water contaminated with *Leptospira* [1]. There are two chromosomes in the *Leptospira* species with a cumulative length ranging from 3.9 to 4.6 Mb. This variability in the genome length confers the bacteria with an ability to live within diverse environments and adapt to a wide range of hosts [3]. Approximately 60% of the functional genes that affect the unique pathogenic mechanisms caused by *Leptospira* are unknown [4].

In 2017, the 100K Pathogen Genome Project was established with internationalization coprojects by many countries, including China, South Korea, and Mexico. This project provides various pathogen draft genomes from many areas, and which include human and animal diseases, food, environmental reservoirs of those pathogens and wildlife. Several species such as *Campylobacter*, *Shigella*, *Salmonella*, *Listeria*, *Helicobacter*, and *Vibrio* are currently involved in the project [5]. Virulence genes code for virulence factors that are essential for successful infection and pathogenesis, such as invasion, colonization, adaptation in host environments, immune evasion and tissue damage. Comparison of genomes from microorganisms causing the variety of symptoms provides insight into the mechanisms of microbial infection and pathogenesis. The virulence factor database (VFDB) [6] provides up-to-date information of virulence factor genes from various bacterial pathogens.

In this study, we compared the genomes of *Leptospira* isolated in Thailand from both mild and severe leptospirosis patients. The data provide insight into the genomic characteristics of *Leptospira interrogans*. In addition, virulence factor genes were analyzed using bioinformatics approaches. This research provides information for therapeutic and vaccine development for leptospirosis.

## Methods

### Isolation of *Leptospira*

*Leptospira* isolated from human patients in this study were obtained from the Department of Medicine, Faculty of Medicine, Siriraj Hospital, Mahidol University, Bangkok, Thailand. The protocol was approved by the Ethical Committee of the Ministry of Public Health, Royal Government of Thailand. One isolate was from a mild leptospirosis patient, while the other was from a patient presenting with a severe clinical manifestation. Leptospirosis was laboratory confirmed by detecting IgM antibody to *Leptopsira* by indirect immunofluorescent assay and PCR for *lipL32* gene detection. Briefly, the mild case (TH_mild) was a 25-year-old male, admitted to Loei Hospital on 21 August 2001. He presented with three days of fever, headache and myalgia. *Leptospira* detected from his blood culture was identified as Serogroup Pyrogenase. The severe case (TH_severe) was a 59-year-old male admitted to Nakhon Ratchasima Hospital on 2 July 2012. He presented with septic shock and died within 48 h of admission. He had a history of 3 days of fever and developed hypotension, jaundice, acute renal failure and upper gastrointestinal hemorrhage. He had no hemoptysis or acute respiratory distress syndrome.

### Library preparation

DNA was extracted from the leptospires grown in EMJH medium using QIAamp DNA mini kit (Qiagen, Valencia, CA, USA) according to the manufacturer’s instructions. In the fragmentation step, a Covaris M220 focused-ultrasonicator (Covaris, Brighton, UK), with 20% duty factor, 50 unit of peak incident power (W), and 200 cycles per burst for 150 s, was used to fragment 1 µg of DNA. In the DNA library preparation, the fragmented DNA was prepared based on the TruSeq DNA LT Sample Prep Kit (Illumina, San Diego, CA, USA) following the manufacturer’s instructions. Then, AMPure XP beads (Beckman Coulter, Danvers, MA, USA) was used to perform clean up and size selection of the DNA library. The concentration of the DNA library was measured using the KAPA Library Quantification Kit (Kapa Biosystems, Wilmington, MA, USA). The DNA library was diluted to 6 pM. Finally, the diluted DNA library was paired-end sequenced (2 × 150 bp) with the MiSeq platform (Illumina), using MiSeq Reagent Kits V2 (300 cycles) according to the standard protocol.

### Quality filter and genome assembly

MiSeq was used to sequence the mild and severe strains of *Leptospira* isolated from the Thai patients. Trimmomatic-0.38 [7] was used to trim and remove low quality reads using default parameter. *De novo* assembly was performed in both strains using SPAdes-3.13.0 [8]. All scaffolds were checked for contamination of 16S rRNA using the ContEST16s database [9]. The Artermis comparison tool (ACT) [10] was used to perform alignment of assembled sequences to a reference genome using *L. interrogans* serovar Lai 56601 as a reference. The DNA sequences were deposited in the Sequence Read Archive data of NCBI server (BioProject PRJNA716760).

### Gene prediction and functional annotation

In the gene prediction step, Prokka 1.13.3 [11] was used to predict genes in the mild and severe *Leptospira* genome. Putative protein coding sequences from Prokka were performed in the functional annotation. The integration of annotation data from the EggNOG database version 1.0.3 [12] and the David gene ontology (GO) database [13] represent the function of predicted genes including the cluster of orthologous groups of proteins (COGs), Kyoto Encyclopedia of Genes and Genomes (KEGG) pathway [14], and GO annotation.

### Prediction of virulence factor gene

The putative protein coding sequences were searched using blastP with the VFDB. The criteria for the determination of candidate virulence sequences was based on an e-value of 10e^-5^. Venn diagram analysis was used to find unique candidate virulence sequences in a specific strain. Lipoprotein prediction in gram-negative bacteria was performed using LipoP 1.0 [15].

### Identification of phages in mild and severe *Leptospira* genomes

PHASTER (PHAge Search Tool Enhanced Release) [16] was performed to identify phages in both the mild and severe genomes.

## Results

### Genome characteristics of mild and severe strain

There was a total of 5,439,790 and 2,162,355 reads with 150 bp paired-end library using mean Phred score (Q) > 30 in mild and severe strain, respectively. The number of scaffolds more than 500 bp are 165 in the mild strain and 309 in the severe strain. The overview of fastq and *de novo* data assembly of mild and severe strains is shown in Table 1. After merging and ordering scaffolds with ACT, there are 3,947 and 297 predicted genes in the final assembly of chromosome 1 (4.70 Mb) and chromosome 2 (0.36 Mb), respectively. In the severe strain, there are 4,373 and 236 predicted genes in the final assembly of chromosome 1 (5.14 Mb) and chromosome 2 (0.37 Mb), respectively. The large variations of the CG content regions in the genome may be caused by being over- or under-fragmented during the library construction. The percentage of GC content in *Leptospira interogans* ranges from 35%‒41% [17]. The mild genome had an average GC content of 35%, and the severe genome had an average GC content of 37%.

**Fig. 1.**
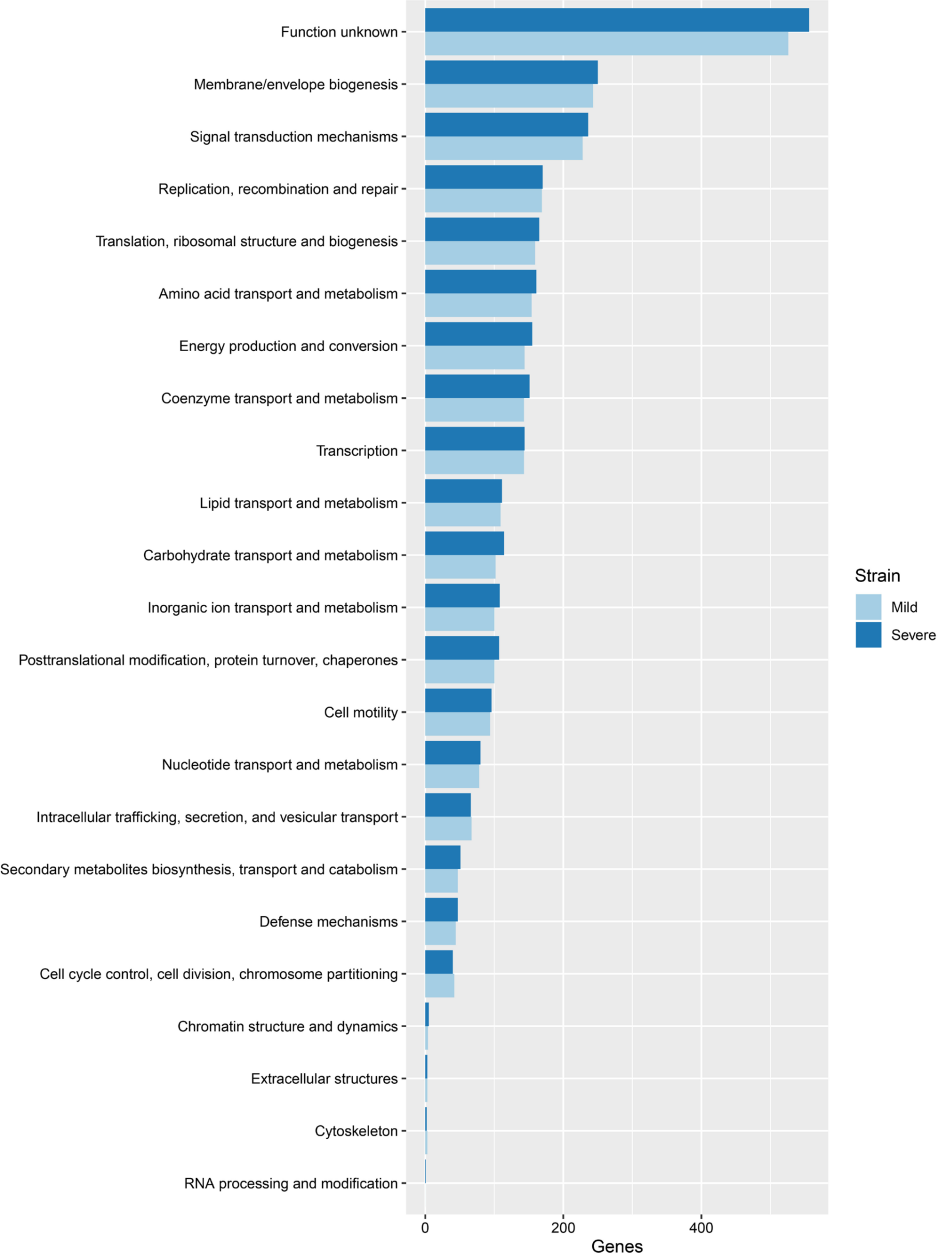
Comparison of clusters of orthologous groups of proteins between mild and severe strains.

From COGs analysis of mild and severe strains, the top three categories included function unknown, membrane/envelope biogenesis and signal transduction mechanisms, as indicated in Fig. 1. For the KEGG pathway analysis, the top three pathways included metabolic pathways, biosynthesis of amino acids, and 2-oxocarboxylic metabolism acid, as shown in Supplementary Fig. 1. Functional annotation is the process of collecting information about the function of genes. The GO system [18] was used in this study. There are three distinct categories in GO, namely molecular function, cellular component and biological process. The results of GO analysis given in Supplementary Figs. 2‒4 show that the top three molecular functions are sigma factor activity, magnesium ion binding, and structural constituent of ribosome. The top three cellular components are cytoplasm, ribosome, and large ribosomal subunit. The top three biological processes are DNA-templated transcription/initiation, translation, and peptidoglycan biosynthetic process. There is no significant difference between mild and severe strains from COGs, KEGG pathway and GO analysis.

### Putative virulence factor analysis

**Fig. 2.**
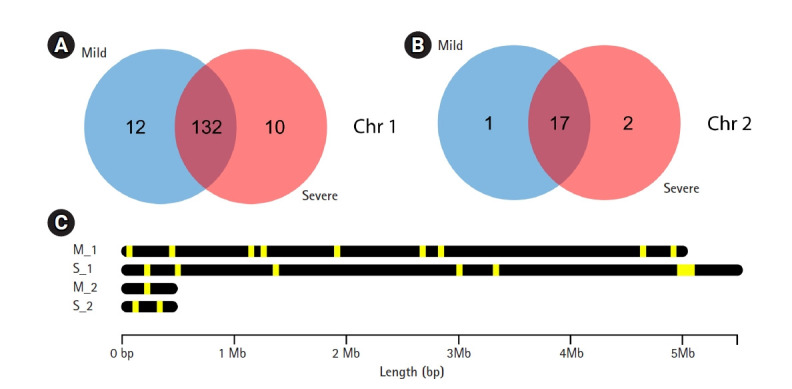
Comparison of virulence factor genes between mild and severe strains. (A) Venn diagram analysis between mild and severe strains in chromosome 1. (B) Venn diagram analysis between mild and severe strains in chromosome 2. (C) Comparison region of predicted virulence factor genes in each chromosome of both mild and severe strains (M_1: chromosome 1 in mild strain, M_2: chromosome 2 in mild strain, S_1: chromosome 1 in severe strain and S_2 chromosome 2 in severe strain; Yellow stripe in the black bar: region of virulence factor genes).

A total of 4,244 and 4,699 predicted genes in mild and severe strains, respectively from Prokka were used to identify virulence factor gene with VFDB. The 162 and 161 virulence factor genes were found in mild and severe stains, respectively using blastP with an e-value < 10e^-5^. Venn diagram analysis was used to compare virulence factor genes between mild and severe strains. Fig. 2A shows that 12 genes and 10 genes, respectively, of chromosome 1 were found in only the mild strain and only the severe strain. In chromosome 2, one gene was found in the mild strain only and two genes were found in the severe strain only (Fig. 2B). The gene lists that were discovered in only the mild strain included *AfaG-VII, neuA/flmD, rhmA, dapH, yhbX, murB, ahpC, flhB, LA_3103, nuc, PS_PT04340, ipaH2.5*, and *rfaK*. Meanwhile, the gene lists found in only the severe strain consist of *mntB, iga, flgG, proC, kdnB, neuA_1, neuA_2, pyrB, C8J_1334, rfbB, gtf1*, and *hemB*. The description of virulence factor genes is shown in Tables 2 and 3. In Fig. 2C, the regions of virulence factor genes were mapped into chromosomes of mild and severe strains. There are many different regions of virulence factor genes found in mild and severe strains, especially in chromosome 1. In chromosome 2 of the severe strain, the group of virulence factor genes were located in the range of 4.8‒5.2 Mb. In addition, nearby virulence factor genes might exhibit co-expression or regulation. However, nearby virulence factor genes will be studied further.

### Phage analysis

For phage investigation, prophage sequences in mild and severe strain genomes were identified and annotated using PHASTER. Prophages play an important role in the evolution of the bacterial host and are commonly found in the bacterial genome [19]. In our results, there is no phage in either mild and severe genomes. However, the size ranges of incomplete phages from 6.9‒11.3 kb were detected in both strains. PHAGE_Synech_S_CAM7_NC_031927, PHAGE_Sphing_PAU_NC_019521, PHAGE_Synech_ACG_2014b_NC_027130, PHAGE_Bacill_Finn_NC_020480, PHAGE_Psychr_pOW20_A_NC_020841 and PHAGE_Shigel_Sf6_NC_005344 were found in the mild genome. Moreover, PHAGE_Acinet_Acj9_NC_014663, PHAGE_Bacill_SP_15_NC_031245, PHAGE_Synech_S_CAM7_NC_031927, PHAGE_Sphing_PAU_NC_019521 and PHAGE_Synech_ACG_2014f_NC_026927 were found in the severe genome. Almost all of the incomplete prophages were similar to other *Leptospira* species that contained incomplete phages with sizes ranging from 4.1 to 13.8 kb [20]. However, PHAGE_Acinet_Acj9_NC_014663 which was found in the severe strain, is the one multiple-drug resistant species [21].

### Plasmid analysis

Additional investigation of plasmids in the TH_mild and TH_severe strains isolated form Thai patients found that both strains contained *L. interrogans* serovar Canicola strain Gui44 plasmids (pGui1 and pGui2), *L. interrogans* serovar Linhai str. 56609 plasmids (lcp1 and lcp2) and *L. interrogans* serovar Manilae strain UP-MMC-NIID LP plasmid pLIMLP1. Interestingly, the *L. borgpetersenii* serovar Ballum strain 56604 plasmid lbp2 was found only in the TH_severe strain, implying that this plasmid might be associated with the pathogenesis or severity of *Leptospira*.

### Lipoprotein analysis

**Fig. 3.**
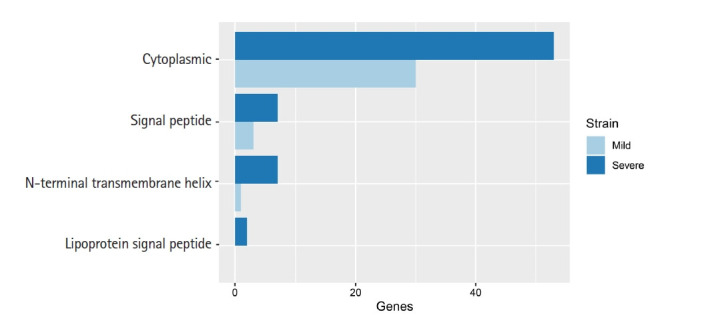
Comparison of lipoprotein predicted genes between mild and severe strains. The class of prediction from LipoP 1.0 was separated into four groups including cytoplasmic, signal peptide, N-terminal transmembrane helix, and lipoprotein signal peptide.

Lipoproteins of bacteria are a set of membrane proteins. There are many functions in the role of pathogenesis and host-pathogen interaction, especially the functions of surface adhesion and initiation of inflammatory processes through translocation of virulence factors in the host cytoplasm [22]. In our study, we used 32 and 67 unique genes in mild and severe strains, respectively, from eggNOG annotation to predict lipoprotein signal peptide using LipoP 1.0. This software can discriminate between lipoprotein and other signal peptides. The prediction was separated into four groups, including cytoplasmic, signal peptide, N-terminal transmembrane helix and lipoprotein signal peptide. In addition, this result in Fig. 3 showed that a protein sequence was assigned to a lipoprotein signal peptide found in the severe strain only.

## Discussion

LipoP1.0 predicts lipoproteins and discriminates between lipoprotein signal peptides and other signal peptides in gram-negative bacteria using a Hidden Markov model (HMM). They report that the accuracy performance of prediction in gram-negative bacteria is 96.8%. Another lipoprotein prediction is called LIPOPREDICT which predicts signal peptides using a support vector machine [23]. The accuracy of this tool is 97%. Support vector machine has a similar performance to HMM. We would like to use LIPOPREDICT to predict lipoproteins in our genomes. Unfortunately, LIPOPREDICT is not available so far.

The genome characteristics of mild and severe strains in this study were compared with the *L. interrogans* genomes previously reported from Russia (strain Taganrog-2018) [24], Sri Lanka (strain FMAS_KW1, FMAS_KW2, and FMAS_AW1) [25], and Saint Kitts (strain SK-1) [26]. The *Leptospira* strains from Russia and Saint Kitts were classified as severe strains. The result of genome characteristics comparison was represented in Supplementary Table 1. In addition, the virulence factor genes were compared among our strains and other strains as shown in Tables 2 and 3. The result revealed that *yhbX, murB, ahpC, flhB*, and *LA_3103* genes were found in *Leptospira interrogans* stains FMAS_KW1, FMAS_KW2, and FMAS_AW1 similar to those found in our mild strain. Moreover, *flgG, proC, neuA_1, neuA_2, pyrB* and *rfbB* genes were also found in *Leptospira interrogans* strains in this study, Taganrog-2018 and SK-1 isolated from severe cases. However, *mntB, iga, kdnB*, and *C8J_1334* genes were found only in our severe strain.

IgA-specific serine endopeptidase or IgA protease is secreted by gram-negative bacteria. This enzyme plays an important role in human antibodies. They can specifically cleave IgA, which provides an antibody for defending the mucosal surface [27]. The inactivation of IgA protease might have the potential to reduce bacterial colonization on mucosal surfaces [28]. Aminoglycosides are broad-spectrum antibiotics that are used in gram-negative and gram-positive organisms [29]. Many reports showed that *Leptospira* are sensitive to aminoglycosides [30,31]. dTDP-glucose-4,6-dehydratase genes were related in a gene cluster in an aminoglycoside antibiotics producer [32].

In bacteria, metal ions play an important role in survival in their host environment. Bacteria which cannot maintain proper homeostasis of metals are less virulent [33]. In many biological processes metal ions are needed as metalloprotein materials, which function as enzyme cofactors or structural elements. Manganese (Mn) is one important example. Many bacteria require manganese with eukaryotic host cells to form pathogenic or symbiotic interactions [34]. Currently, there is evidence that the invading microbe uses Mn as the main micronutrient to avoid the effects of host-mediated oxidative stress and thus plays a significant role in the human host's tolerance to pathogenic bacteria [35]. In our study, we found manganese transport system membrane protein MntB (*mntB*) in the severe *Leptospira* strain. This gene encodes transmembrane protein. The *mntB* gene is part of the ABC transporter system for manganese that mediates the movement of various substrates from microbes to humans across different biological membranes [36]. The lack of the *mntB* gene might affect the homeostasis of metal in bacteria that are less virulent.

The flagellum consists of three main sections, including a flagellar filament, a hook complex, and a basal body in both gram-negative and gram-positive bacteria. There are many genes related to flagellar biosynthetic protein such as *flhA, flhB* [37,38]. The results showed that *flhB* was found in the mild strain. This result came from blastP with a VFDB. However, *flhB* was also found in the severe strain from Prokka annotation. In this case, some genes in the mild strain are similar to the *flhB* gene in other species of bacteria in the VFDB.

In this study, two strains of *Leptospira* spp. isolated from mild and severe Thai patients were compared. Our analysis showed 3,947 and 297 predicted genes in the final assembly of chromosome 1 (4.70 Mb) and chromosome 2 (0.36 Mb), respectively, in the mild strain. In addition, there are 4,373 and 236 predicted genes in the final assembly of chromosome 1 (5.14 Mb) and chromosome 2 (0.37 Mb), respectively, in the severe strain. The difference of virulence factor genes was found in both strains. Our results focus on predicting virulence factor genes in the severe strain that is not found in the mild strain. The virulence factor genes in the severe strain are only related to host immune response, and survival in the host environment might be the vital virulence factor genes. However, these genes should be validated in further study.

## Figures and Tables

**Table 1.** Characteristics of mild and severe data and *de novo* assembly

Feature	Mild	Severe
Length (bp)	150	150
Raw reads	5,989,479	2,590,133
Q30 reads	5,439,790	2,162,355
No. of scaffolds	619	1,210
No. of scaffolds (>500 bp)	165	309
N50	97,013	185,969

**Table 2.** Description of predicted virulence factor genes in mild strains

Gene	Description	TH_mild	FMAS_KW1	FMAS_KW2	FMAS_AW1
*AfaG-VII*	Afimbrial adhesin	√	X	X	X
*neuA/flmD*	CMP-N-acetylneuraminic acid synthetase	√	X	X	X
*rhmA*	2-Keto-3-deoxy-L-rhamnonate aldolase	√	X	X	X
*dapH*	2,3,4,5-Tetrahydropyridine-2,6-dicarboxylate N-acetyltransferase	√	X	X	X
*yhbX*	Outer membrane protein YhbX	√	√	√	√
*murB*	UDP-N-acetylenolpyruvoylglucosamine reductase	√	√	√	√
*ahpC*	Alkyl hydroperoxide reductase C	√	√	√	√
*flhB*	Flagellar biosynthetic protein FlhB	√	√	√	√
*LA_3103*	Fibronectin-binding protein	√	√	√	√
*nuc*	Thermonuclease	√	X	X	X
*PS_PTO4340*	Insecticidal toxin protein, putative	√	X	X	X
*ipaH2.5*	Invasion plasmid antigen	√	X	X	X
*rfaK*	Alpha 1,2 N-acetylglucosamine transferase	√	X	X	X

**Table 3.** Description of predicted virulence factor genes in severe strains

Gene	Description	TH_severe	Taganrog-2018	SK-1
*mntB*	Manganese transport system membrane protein MntB	√	X	X
*iga*	IgA-specific serine endopeptidase	√	X	X
*flgG*	Flagellar basal-body rod protein FlgG	√	√	√
*proC*	Pyrroline-5-carboxylate reductase	√	√	√
*kdnB*	3-Deoxy-alpha-D-manno-octulosonate 8-oxidase	√	X	X
*neuA_1*	N-Acylneuraminate cytidylyltransferase	√	√	√
*neuA_2*	CMP-N,N'-diacetyllegionaminic acid synthase	√	√	√
*pyrB*	Aspartate carbamoyltransferase catalytic subunit	√	√	√
*C8J_1334*	Hypothetical protein	√	X	X
*rfbB*	dTDP-glucose 4,6-dehydratase	√	√	√
*gtf1*	Glycosyltransferase Gtf1	√	X	√
*hemB*	Delta-aminolevulinic acid dehydratase	√	X	√

## Supplementary Materials

Supplementary Table 1. Comparison genome characteristics of Leptospira interrogans genomes

Supplementary Fig. 1. Comparison of KEGG pathway between mild and severe strains.

Supplementary Fig. 2. Comparison of molecular function between mild and severe strains.

Supplementary Fig. 3. Comparison of cellular component between mild and severe strains.

Supplementary Fig. 4. Comparison of biological process between mild and severe strains.
