# Supplementary material for: Comparative genome characterization of Leptospira interrogans from mild and severe leptospirosis patients
Source: Genomics Inform. 2021 Sep 30;19(3):e31. doi: 10.5808/gi.21037 (PMC8510873; doi:10.5808/gi.21037)
Supplement: Supplementary Table 1. — Comparison genome characteristics of Leptospira interrogans genomes [file gi-21037suppl1.pdf]

Supplementary Table 1. Comparison genome characteristics of *Leptospira interrogans* genomes

Genome	Size (Mb)	Gene	Accession number	Reference
Leptospira interrogans strain Taganrog-2018	4.50	3,974	SJDW00000000.1	[1]
Leptospira interrogans strain FMAS_KW1	4.67	3,823	CP039258.1 and CP039259.1	[2]
Leptospira interrogans strain FMAS_KW2	4.65	3,836	CP039256.1 and CP039257.1	[2]
Leptospira interrogans strain FMAS_AW1	4.86	4,034	CP039283.1 and CP039284.1	[2]
Leptospira interrogans strain SK-1	4.63	3,731	CP048830 and CP048831	[3]
Leptospira interrogans strain TH_mild	5.06	4,244	CP075694 and CP075695	In this study
Leptospira interrogans strain TH_severe	5.51	4,609	CP075692 and CP075693	In this study

TH_mild is the *Leptospira interrogans* mild strain. TH_severe is the *Leptospira interrogans* severe strain.

References

1. Samoilov AE, Stoyanova NA, Tokarevich NK, Evengard B, Zueva EV, Panferova YA, et al. Lethal outcome of leptospirosis in southern Russia: characterization of *Leptospira interrogans* isolated from a deceased teenager. Int J Environ Res Public Health 2020;17:4238.

2. Senevirathna I, Jayasundara D, Lefler JP, Chaiboonm KL, Warnasekara J, Agampodi S, et al. Complete genome sequence of *Leptospira interrogans* strains FMAS_KW1, FMAS_KW2 and FMAS_AW1 isolated from leptospirosis patients from Karawanalla and Awissawella, Sri Lanka. J Genomics 2020;8:49-52.

3. Llanes A, Prakoso D, Restrepo CM, Rajeev S. Complete genome sequence of a virulent *Leptospira interrogans* serovar Copenhageni strain, assembled with a combination of nanopore and Illumina reads. Microbiol Resour Announc 2020;9:e00200-20.
