# Supplementary figures and images for: Comparative genome characterization of Leptospira interrogans from mild and severe leptospirosis patients
Source: Genomics Inform. 2021 Sep 30;19(3):e31. doi: 10.5808/gi.21037 (PMC8510873; doi:10.5808/gi.21037)

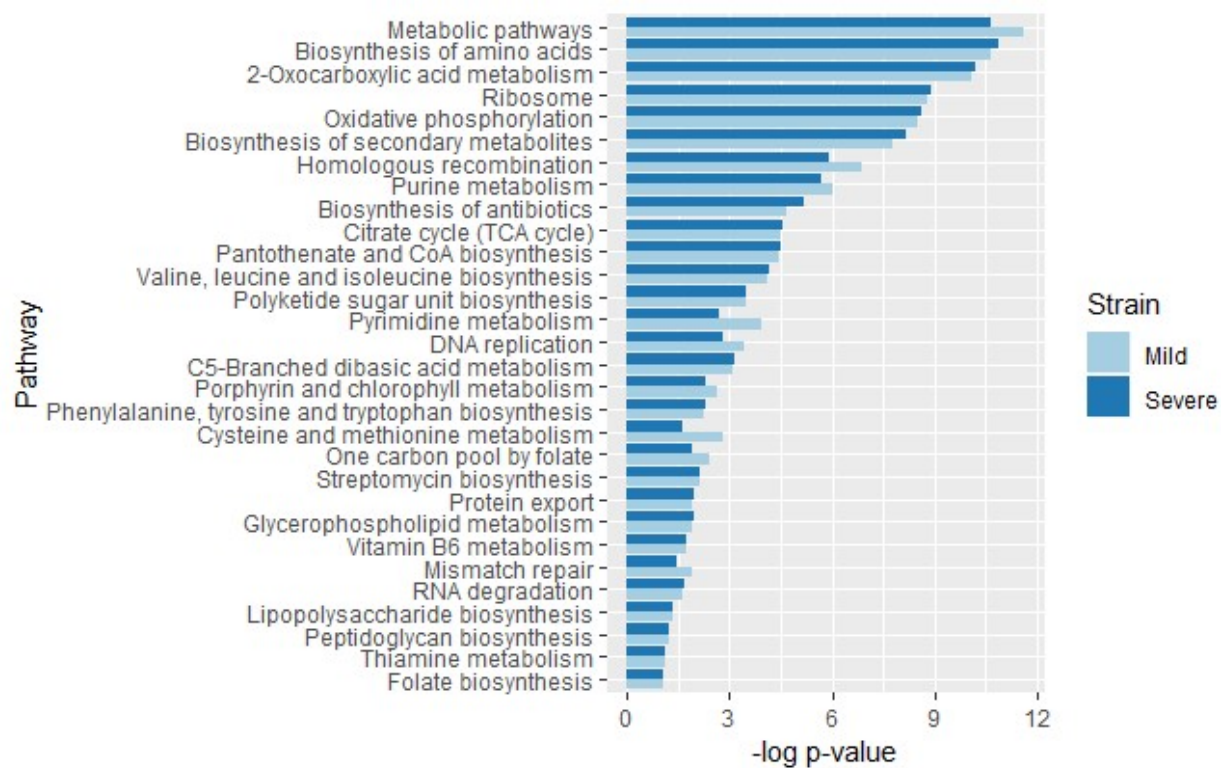

**Supplementary Fig. 1.** Comparison of KEGG pathway between mild and severe strains.

Supplement: Supplementary Fig. 1. — Comparison of KEGG pathway between mild and severe strains. [file gi-21037suppl2.pdf]

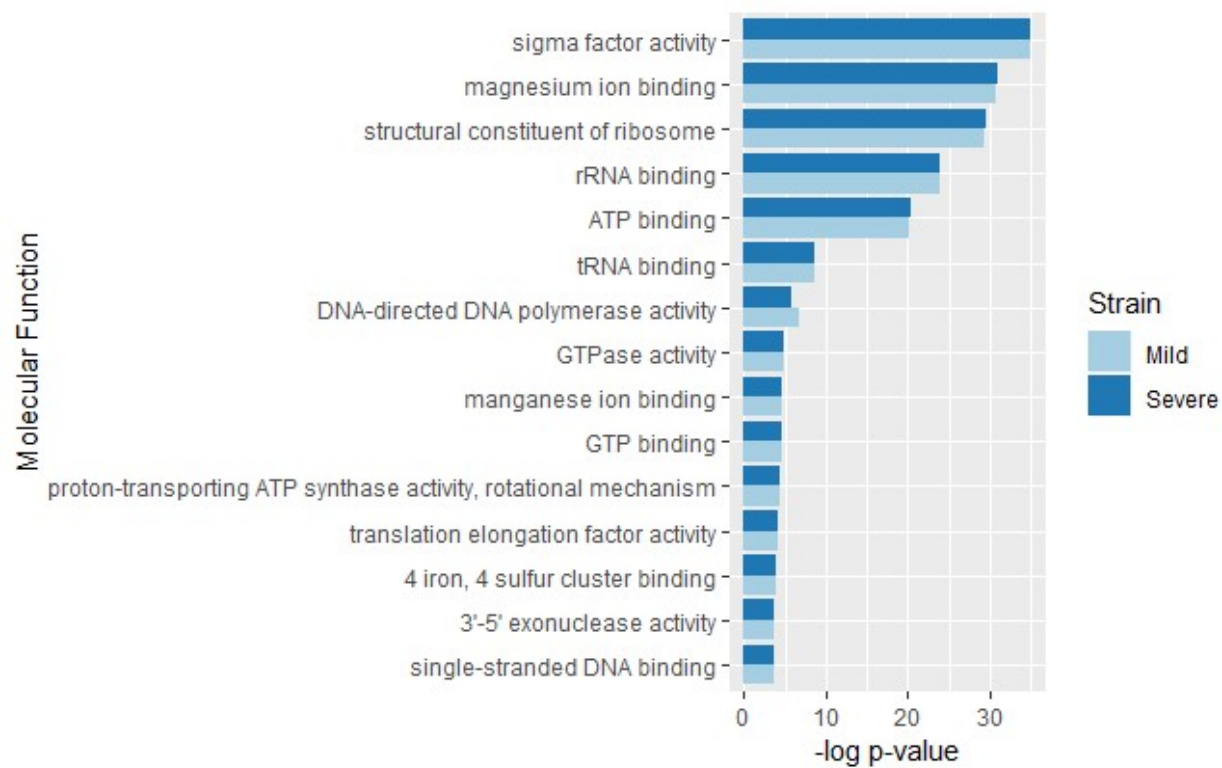

**Supplementary Fig. 2.** Comparison of molecular function between mild and severe strains.

Supplement: Supplementary Fig. 2. — Comparison of molecular function between mild and severe strains. [file gi-21037suppl3.pdf]

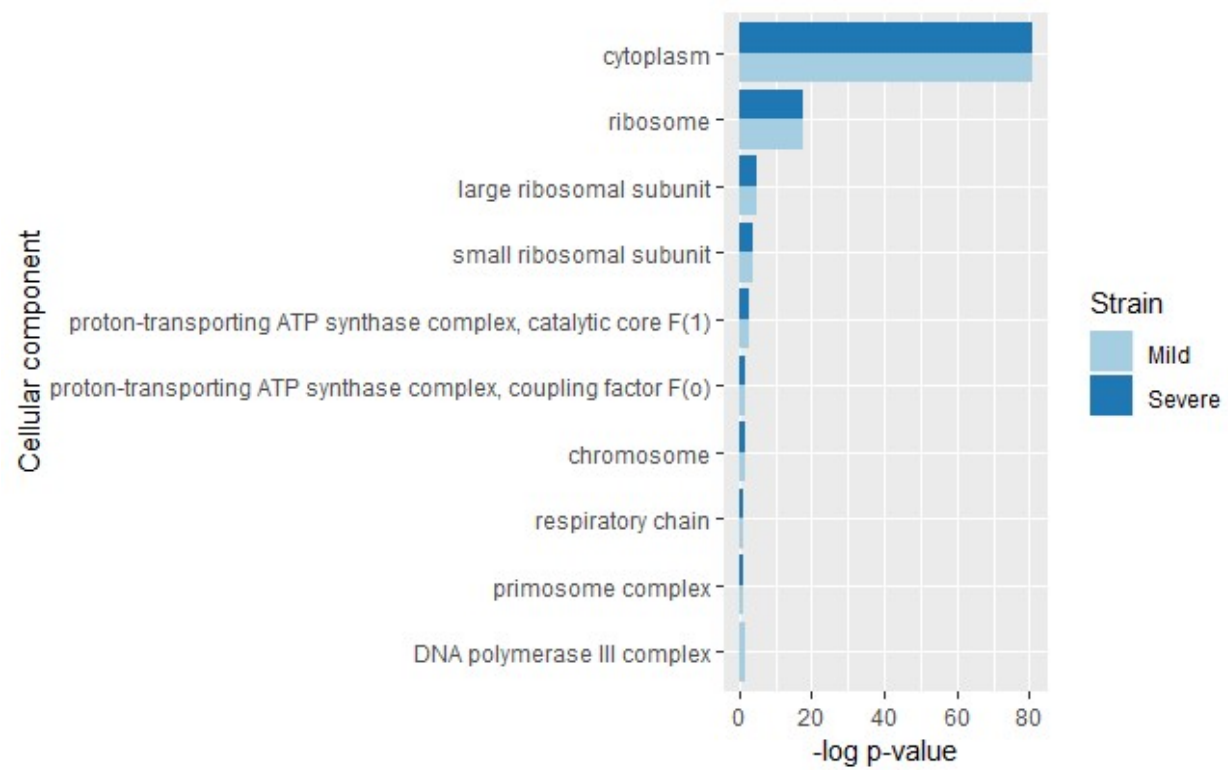

**Supplementary Fig. 3.** Comparison of cellular component between mild and severe strains.

Supplement: Supplementary Fig. 3. — Comparison of cellular component between mild and severe strains. [file gi-21037suppl4.pdf]

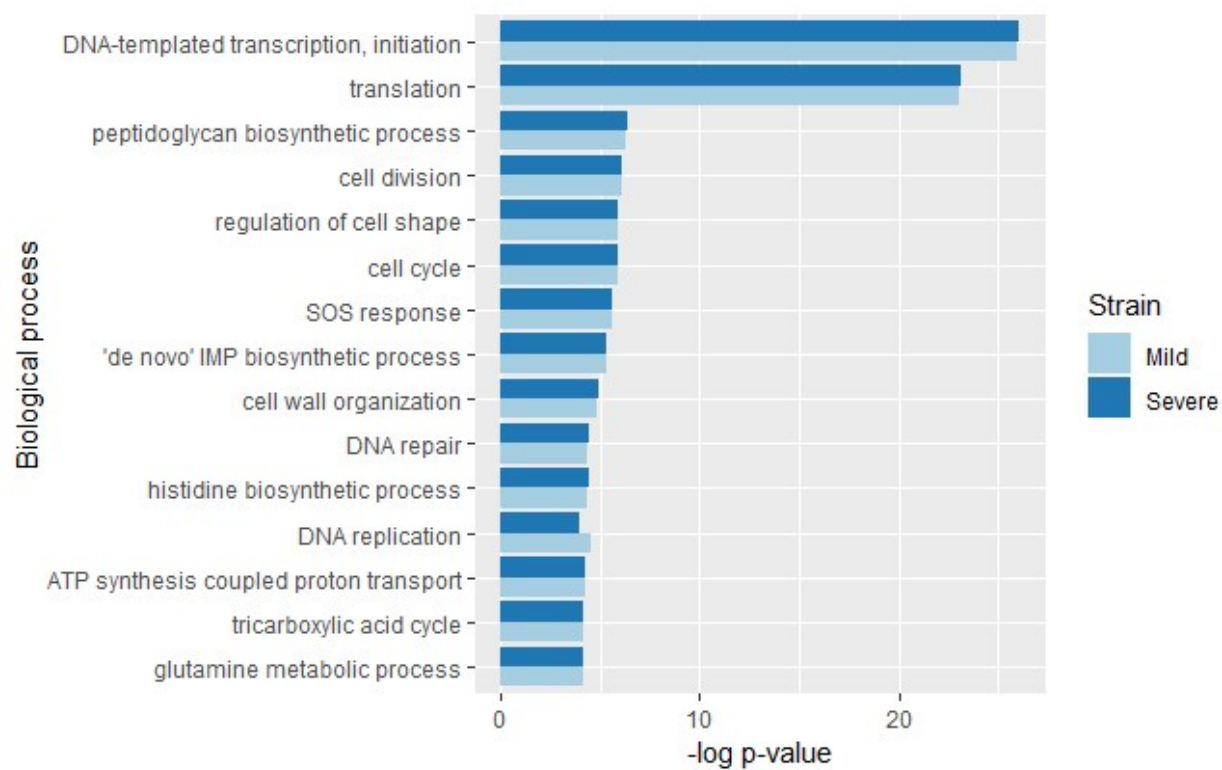

**Supplementary Fig. 4.** Comparison of biological process between mild and severe strains.

Supplement: Supplementary Fig. 4. — Comparison of biological process between mild and severe strains. [file gi-21037suppl5.pdf]
